# Sex specific effects of pre-pubertal stress on hippocampal neurogenesis and behaviour

**DOI:** 10.1038/s41398-018-0322-4

**Published:** 2018-12-10

**Authors:** Nichola Marie Brydges, Anna Moon, Lowenna Rule, Holly Watkin, Kerrie L. Thomas, Jeremy Hall

**Affiliations:** 1 Neuroscience and Mental Health Research Institute, Cardiff University, Hadyn Ellis Building, Maindy Road, Cardiff, CF24 4HQ UK; 2 School of Biosciences, Cardiff University, Museum Avenue, Cardiff, CF10 3AX UK; 3 MRC Centre for Neuropsychiatric Genetics and Genomics, Cardiff University, Hadyn Ellis Building, Maindy Road, Cardiff, CF24 4HQ UK

## Abstract

Experience of traumatic events in childhood is linked to an elevated risk of developing psychiatric disorders in adulthood. The neurobiological mechanisms underlying this phenomenon are not fully understood. The limbic system, particularly the hippocampus, is significantly impacted by childhood trauma. In particular, it has been hypothesised that childhood stress may impact adult hippocampal neurogenesis (AHN) and related behaviours, conferring increased risk for later mental illness. Stress in utero can lead to impaired hippocampal synaptic plasticity, and stress in the first 2–3 weeks of life reduces AHN in animal models. Less is known about the effects of stress in the post-weaning, pre-pubertal phase, a developmental time-point more akin to human childhood. Therefore, we investigated persistent effects of pre-pubertal stress (PPS) on functional and molecular aspects of the hippocampus. AHN was altered following PPS in male rats only. Specifically males showed reduced production of new neurons following PPS, but increased survival in the ventral dentate gyrus. In adult males, but not females, pattern separation and trace fear conditioning, behaviours that rely heavily on AHN, were also impaired after PPS. PPS also increased the expression of parvalbumin-positive GABAergic interneurons in the ventral dentate gyrus and increased glutamic acid decarboxylase 67 expression in the ventral hilus, in males only. Our results demonstrate the lasting effects of PPS on the hippocampus in a sex- and time-dependent manner, provide a potential mechanistic link between PPS and later behavioural impairments, and highlight sex differences in vulnerability to neuropsychiatric conditions after early-life stress.

## Introduction

Childhood adversity has repeatedly been identified as a risk factor for mental and physical illness^1^. However, neurobiological mechanisms underpinning this relationship are not fully understood. Stressful experiences activate the hypothalamic-pituitary-adrenal (HPA) axis, resulting in the release of corticosteroid hormones. These hormones traverse the blood–brain barrier, providing a pathway through which stress can alter brain development and leave individuals susceptible to a range of psychiatric disorders later in life. The limbic system, especially the hippocampus, is predicted to display considerable sensitivity to stress during early life; it contains high levels of glucocorticoid receptors, and is undergoing significant structural and functional changes as it matures^2,3^. Accordingly, childhood maltreatment is consistently associated in humans with reduced hippocampal volume, particularly in the dentate gyrus and Cornu Ammonis 3 (CA3) subfields^1,4–7^. Hippocampal-dependent behaviours, such as pattern recognition, spatial memory and verbal declarative memory, are also impaired in human sufferers of childhood maltreatment^8–10^. Although informative, studies in humans do not allow extensive investigation into the underlying mechanisms of the impact of early-life stress on the hippocampus to be explored.

In rodents, stress early in life can produce significant alterations in the adult hippocampus. Stress in utero can lead to impaired hippocampal synaptic plasticity, and stress in the first 2–3 weeks of life reduces adult hippocampal neurogenesis (AHN) in the dentate gyrus and brain-derived neurotrophic factor expression^11–13^. Less is known about the effects of stress in the post-weaning, pre-pubertal phase, a developmental time-point more akin to human childhood^14^. Studies to date have revealed that pre-pubertal stress (PPS) impairs synaptic plasticity in the dorsal hippocampus, alters the expression of corticosteroid receptors and impairs performance in a hippocampal-dependent contextual fear task in males^15–17^.

AHN is believed to play a crucial role in a number of hippocampal-dependent cognitive processes, including contextual fear responses, trace fear conditioning and pattern separation^18–21^. Pattern separation is a computational process that keeps similar input patterns distinct, allowing similar memories to remain separate^22^. The more comparable the memories, the harder it is to ‘pattern separate’ them. Experimentally, pattern separation can be assessed through analysing the ability to spatially discriminate between objects placed relatively far apart (easy condition, spatial locations do not overlap greatly) or close together (difficult condition, spatial overlap and potential for interference greater)^20,21,23^. This function is attributed to the dentate gyrus, through the unique action of young adult-born neurons^20–22,24,25^. These neurons also play a specific role in fear conditioning—reductions in AHN using either antimitotic agents or transgenic models specifically impairs fear learning only when a delay is interposed between the fear-associated and aversive stimuli, known as trace conditioning^26–28^. AHN is exquisitely sensitive to a range of environmental factors, notably stress, physical exercise and environmental enrichment^19^. Therefore, the first aim of this study was to determine whether PPS would decrease AHN and impair behaviours that rely on AHN, specifically, pattern separation and trace conditioning.

A number of intracellular pathways and extrinsic factors regulate AHN^29,30^. GABA signalling plays a particularly important role in AHN, regulating division of neural stems cells and modulating survival of newborn neurons^31,32^. Therefore, the second aim of this study was to investigate the consequences of PPS on parvalbumin (PV) + GABAergic (γ-aminobutyric acid) interneurons and glutamic acid decarboxylase 67 (GAD67) expression to elucidate a mechanistic link between PPS and adult hippocampal function.

## Methods and materials

### Animals

Animal experiments adhered to the European regulations on animal experimentation (Directive 2010/63/EU) and the UK Home Office Animals (Scientific Procedures) Act 1986. Male and female Lister-hooded rats were bred in-house at Cardiff University from adult pairs (Charles River) for this study. Litters were weaned on postnatal day 21 (PND 21), and housed in same-sex cages (32 cm × 50 cm × 21 cm) with littermates. Males and females were housed in the same room, food and water were provided ad libitum unless stated otherwise, cages were lined with wood shavings, a cardboard tube and wooden stick were provided as enrichment and light was maintained on a 12:12-h light/dark cycle (not reverse). Between 9 and 13 animals per group and sex were used in each experiment, and separate cohorts of animals were used for each behavioural experiment and for immunohistochemistry. Sample sizes were based on power calculations from previous studies using the same animal model and similar behavioural analyses^16,17^.

### Pre-pubertal stress

On PND 25–27, half of the litters experienced a short-term PPS protocol that has been described previously^16,33,34^, and was originally described by Jacobson-Pick and Richter-Levin^35^. This took place in a designated room, separate from the holding room. On PND 25, between 9:00 a.m. and 13.00 p.m., animals were given a 10 min swim stress in an opaque swimming tank (25 cm high, 34 cm diameter), 12 L capacity filled with 6 L of 25 ± 1 °C water. PND 26 involved restraint stress: between 9:00 a.m. and 13:00 p.m. animals were placed into plastic restraint tubes (15 cm length, 5 cm diameter) for three sessions of 30 min, separated by 30 min breaks in the home cages. The final stressor on PND 27 involved placing animals onto an elevated platform (15 × 15 cm^2^, 115 cm high) for three 30-min sessions, separated by 60 min in the home cage between 9:00 a.m. and 5:00 p.m. Animals were then returned to their home cages and holding rooms and left undisturbed (aside from cage cleaning) until early adulthood (PND 60).

Litters were alternately allocated to experimental groups (PPS or control) by order of birth. A maximum of three animals per litter were used in each experiment, and a minimum of five litters per group (PPS or control) were generated to minimise effects of pseudoreplication^36^. The litter of origin was accounted for in all statistical analyses.

### BrdU administration

Forty-four rats (male: 10 control, 13 PPS; female: 10 control, 11 PPS) were injected intraperitoneally on PND 60–65 with bromodeoxyuridine (BrdU, 200 mg/kg in 0.9% sterile saline solution) and killed by transcardial perfusion with 0.01 M phosphate-buffered saline (PBS) and 4% paraformadelhyde (PFA) under anaesthesia 24 h later to assess baseline rates of cell proliferation and neurogenesis in the dentate gyrus. Brains were left in PFA overnight (4 °C), and transferred to 30% sucrose solution for cryoprotection. Coronal 30-µm sections were cut through the entire hippocampal extent on a freezing microtome (Leica RM2245) and placed into a solution of cryoprotectant for storage at −20 °C until immunohistochemical analysis.

### Immunohistochemisty

Sections were stained for either: (i) BrdU and doublecortin (DCX), (ii) parvalbumin and DCX or (iii) GAD67.

#### BrdU and DCX

Sections were washed between each step for 3 × 5 min in 0.01 M Tris-buffered saline (TBS, pH 7.4) and all steps were carried out at room temperature, unless otherwise specified. One in every 12 sections was denatured in 45 °C, 1 M HCl for 30 min, followed by incubation in blocking solution (0.3% Triton-X in 0.01 M TBS (TTBS), 2% donkey serum) for 60 min, rat anti-BrdU (1:100 in blocking solution, OBT0030, ABD Serotec, UK) for 48 h at 4 °C and Alexa Fluor 488 (5 µg/ml in TTBS, donkey anti-rat, Life Technologies, UK) for 2 h in the dark. Sections were then incubated with goat anti-DCX (1:100 in blocking solution, SC8066, Santa Cruz Biotechnology, UK) for 24 h at 4 °C to identify newly differentiated immature neurons, Alexa Fluor 647 (10 µg/ml in TTBS, donkey anti-goat, Life Technologies, UK) for 2 h in the dark. Sections were then exposed to DAPI (4′,6-diamidino-2-phenylindole; 1:3000 in TBS, D9542, Sigma UK) for 5 min. Washed sections were then mounted onto glass microscope slides and coverslipped with fluorescence mounting medium (S3023, Dako, UK). The number of cells double labelled with BrdU and DCX were counted in the supra- and infrapyramidal blades of each dentate gyrus in the dorsal (Bregma −1.72 to −5.28 mm) and ventral (Bregma −5.28 to −6.72 mm) portions of the hippocampus according to the Atlas of Paxinos and Watson (2009). The total number of double-labelled cells was estimated by multiplying the total number counted for each area (dorsal or ventral, supra- or infrapyramidal blade) by 12^37^. Counts and total volume of the hippocampus was analysed using Zen Blue (Carl Zeiss Microscopy, Germany).

#### Parvalbumin and DCX

All steps were carried out at room temperature, unless otherwise specified. One in every 12 sections was blocked in blocking solution (1% Triton-X in 0.01 M PBS, 10% donkey serum) for 2 h, goat anti DCX (1:100, SC8066, Santa Cruz) and mouse anti-parvalbumin (1:1000, P3088, Sigma-Aldrich) in 0.1% Triton-X, and 0.2% donkey serum in 0.01 M PBS overnight at 4 °C. Sections were washed in 0.1% Triton-X, 0.2% donkey serum in 0.01 M PBS at least three times before incubation with donkey anti-goat Alexa 647 (10 µg/ml in 0.01 M PBS, Life Technologies UK) and donkey anti-mouse Alexa 555 (1:1000 in 0.01 M PBS, Life Technologies UK) for 2 h in the dark. Sections were washed with 0.01 M PBS and incubated for 10 min in the dark with DAPI stain (1:3000 in 0.01 M PBS, D9542, Sigma UK) for nuclei staining. Sections were washed twice more in 0.01 M PBS before being mounted on standard microscopy slides using Mowiol aqueous mounting medium (Sigma, UK) and standard cover slips. Sections were imaged using an epifluorescent microscope (Leica DM6000B Upright Timelapse System with Leica Application Suite Advanced Fluorescence 3.0.0 build 8134 software, Leica Microsystems). The number of cells were quantified by manual counting in the supra- and infrapyramidal blades of the dorsal (−2.28 to – 5.04 mm) and ventral (−5.16 to −6.48 mm) dentate gyrus. The area was calculated for each region and the number of immunopositive cells quantified through visual counting, giving a cell count per mm^2^. The analysis was performed using Image J, with the plugin ‘Cell Counter’.

#### GAD67

All steps were carried out at room temperature, unless otherwise specified. Sections were washed between each step for 3 × 30 min in blocking solution (0.2% Triton-X in 0.01 M PBS (TTBS) containing 5% donkey serum, 2.5% bovine serum albumin). One in every 12 sections was incubated in blocking solution for 160 min, GAD67 antibody (1:500 in blocking solution, MAB5406, Millipore, UK) for 48 h at 4 °C and Alexa 647 (1:1000 in blocking solution, donkey anti-mouse, Life Technologies, UK) for 150 min in the dark. Sections were then placed into DAPI (1:200 in 0.01 M PBS, D9542, Sigma, UK) for 10 min in the dark before being mounted onto glass slides as above. The number of cells labelled with GAD67 were counted through the entirety of the dentate gyrus (infra- and suprapyramidal blades), CA1, CA2 and CA3 and hilus in the ventral (Bregma 5.28 to −6.72 mm) and dorsal (Bregma −1.72 to −5.28 mm) portions of the hippocampus, according to the Atlas of Paxinos and Watson (2009). The number of cells per measured area was obtained using Zen Blue (Zeiss).

All slides were imaged at ×20 using Axio Scan Z1 (Zeiss). Spearman's *ρ* was used to investigate correlations between PV+, DCX+ and BrdU+ cells.

### Behaviour

#### Pattern separation

##### Animals

Pattern separation ability was assessed using a modified spontaneous location recognition paradigm^20,21^. Forty two rats (male: 11 control, 10 PPS; female: 12 control, 9 PPS) were food restricted to 85–90% of their free feeding weight 1 week before testing (PND 60–100). Animals were handled daily for 5 min during this time.

##### Apparatus

Testing took place in a black plastic circular arena (45 cm high x 75 cm diameter) with wood shavings covering the floor. The arena was placed in the centre of a dimly lit room, and three proximal spatial cues were placed equidistant around the arena to aid spatial orientation. Objects used were tin cans (7.5 cm × 7.5 cm × 11 cm) and small beer bottles (5.5 cm × 5.5 cm × 15 cm) with the labels removed. Blu-tack was used to fix them to the floor of the arena, and they and the arena were cleaned with 70% ethanol solution between trials. A video camera was suspended directly above the arena and trials were recorded for later analysis.

##### Behaviour

**Fig. 1:**
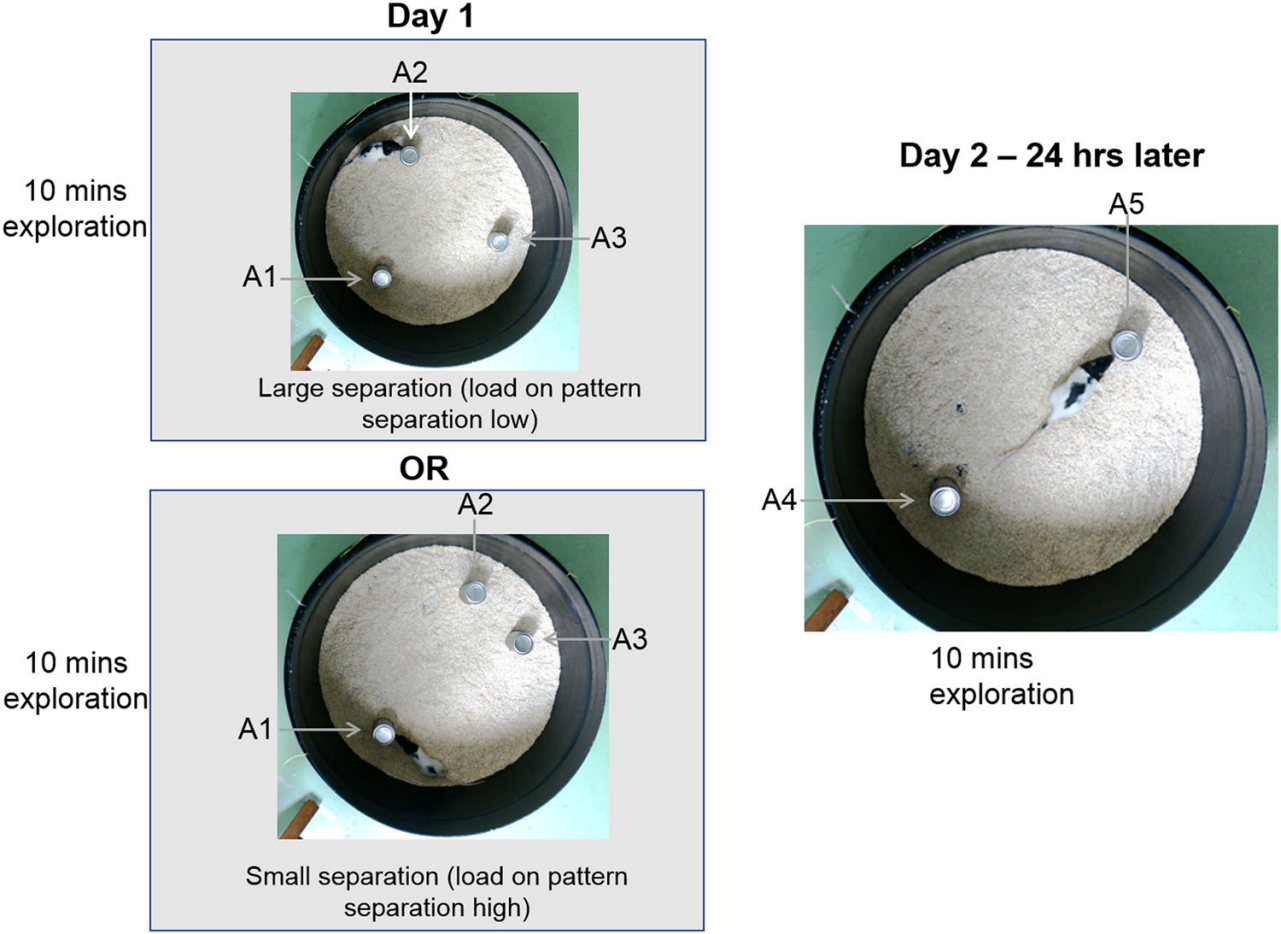
Apparatus used for pattern separation. Animals explored three objects, A1, A2 and A3, in a circular 75 cm arena for 10 min on day 1. The objects were placed 12.5 cm from the perimeter. A2 and A3 were either separated by a **a** large (52 cm) or **b** small distance (20.5 cm). **c** After 24 h, animals were re-exposed to the arena, A1 was replaced with an identical object, A4 and objects A2 and A3 were replaced with A5, placed equidistance to the position of A2 and A3

Animals freely explored the arena for 10 min a day for 5 days. On day 6, objects were placed into the arena. Objects were tin cans for half of the animals from each group and sex, and bottles for the remaining animals. Animals were tested in two conditions—easy (large spatial separation, LS) and difficult (small spatial separation, SS). Three identical objects, A1, A2 and A3 were placed 12.5 cm away from the walls of the arena, 25 cm from the centre with either A2 and A3 at 120° (LS) or 50° (SS) apart (Fig. 1). Animals explored the arena for 10 min (sample phase). After 24 h, animals were returned for 5 min (choice phase). The arena now contained two new objects, A4 and A5, identical in appearance to those used in the sample phase. A4 was in the same (familiar) location as A1, and A5 was placed equidistant to the previous locations of A2 and A3 (novel spatial location, Fig. 1). Rodents display an innate desire to explore novel objects and objects in novel locations over familiar ones^20,38^, thus animals will explore A5 in preference to A1. It will be harder to recognise A5 in the novel location in the SS condition than in the LS condition because SS places greater demand on pattern separation abilities than LS^20,21^. The amount of time animals spent exploring objects in the sample and choice phases was assessed from video recordings by an observer blind to the group. A discrimination ratio was then calculated^20,38^:

$$\frac{{\left( {{\mathrm{time}}\,{\mathrm{exploring}}\,{\mathrm{novel}}\,{\mathrm{location}}\,{\mathrm{object}} - {\mathrm{time}}\,{\mathrm{exploring}}\,{\mathrm{familiar}}\,{\mathrm{location}}\,{\mathrm{object}}} \right)}}{{{\mathrm{total}}\,{\mathrm{exploration}}\,{\mathrm{time}}}}.$$

Half of the animals from each group and sex were tested in the LS first then the SS, the other half experienced SS then LS. Exploration was counted as the rat having its head directed at the object (2 cm or less), or touching the object with its nose. Sitting on or climbing over the object was not counted.

#### Fear conditioning

##### Animals

Animals were trained in trace, delay or control protocols at PND 60–100. One hundred and thirty rats (male: 33 control, 36 PPS; female: 30 control, 31 PPS) were used, with sample sizes of 9–13 per protocol per sex. Three days before testing, animals were handled daily and taken to the testing room for habituation to transport and handling.

##### Apparatus

Testing took place in two identical standard modular test chambers (32 cm × 25.5 cm × 27 cm, Sandown Scientific, UK) with grid floors (19 stainless-steel rods, 1 cm apart) underneath which was a stainless-steel pan. The ceiling, front and back walls of the boxes were clear plexiglass, the sides walls stainless steel, and the chamber was enclosed within a sound-attenuating chamber with a ventilation fan providing a background noise of 63 dB and a video camera attached to the inside of the door. The grid floor was connected to a shock generator, and there was a speaker attached to the inside of the sound-attenuating chamber. The boxes were connected to a computer and shock, light, sound and video generation were controlled by a computer. One box (context one, C1) was scented with a drop of lavender oil (Botanics Aromatherapy Pure Essential Oil, Boots, UK) placed onto a tissue in the pan, and contained an infrared (IR) light bar to allow filming as this box was always dark. In the second box (context two, C2), the pan was filled with wood shavings and the walls and ceiling decorated with black stars on a white background, and this box was always light (via houselight in the test chamber). Boxes were cleaned with ethanol wipes between animals, and sawdust/lavender scent replaced. Half of the animals from each group and sex were trained in C1 and the other half in C2, and all trials were video recorded for later analysis. The conditioned stimulus (CS) was 15 s, 75 dB white noise and the unconditioned stimulus (US) was 0.5 s, 0.5 mA scrambled footshock in all protocols.

##### Behavior

Training. Animals were placed individually into chambers for 120 s. Animals were then trained in one of three protocols: trace protocol (TP)—rats received 10 CS-US pairings, with a 30 s stimulus free trace interval between the offset of the CS and the onset of the US, delay protocol (DP)—10 CS-US pairings were presented, here the CS was followed immediately by the US and control protocol (CP)—animals received 10 presentations of the CS and 10 presentations of the US, which were explicitly unpaired. Intertrial intervals (ITI) were 312s (+/– 62 s) for trace and delay protocols, 156s (+/– 31s) for the control protocol. ITI length is positively correlated with freezing to later presentations of the CS in trace conditioning, and negatively associated with freezing to a context^39^. This length of ITI was selected as optimal for assessing freezing to cue and context in both trace and delay protocols^39^.

Context recall 1. Animals were returned to their training chamber 24 h later for 10 min to assess contextual fear responses.

Cue recall. Forty-eight hours after training, animals trained in C1 were placed into C2 and vice versa to reduce contextual associations and maximise responding to the CS. A plastic insert was placed over the bars of the floor to aid the discrimination. Animals received a 120 s acclimation period, followed by 360 s of CS the 240 s post -CS.

Context recall 2. Seventy-two hours after training, animals were returned to their training chamber for 10 min to assess contextual fear responses.

Freezing during training (baseline, post -shock and trace interval) and recall was analysed from video recordings by an observer blind to group. Freezing was defined as immobility with the exception of movement required for respiration, and was sampled every 10 s. 

### Statistical analysis

All data were checked for homogeneity of variance and normality, and transformed when these conditions were not met. Data were analysed in JMP (statistical software, SAS Institute, Cary, NC, USA) using generalised linear models, with experimental treatment (control or PPS), sex, separation distance (pattern separation only), protocol (trace, delay or control, fear conditioning only) fitted as factors. Litter was nested within group and added as a random factor to account for litter of origin and animal was nested within litter and added as a random factor to account for multiple measurements on the same animal.

## Results

### Neurogenesis

**Fig. 2:**
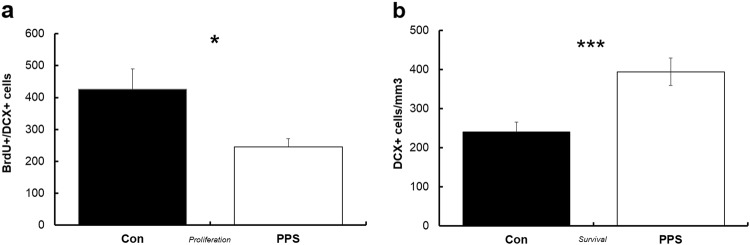
Effects of PPS on neurogenesis. **a** In males, PPS decreased production of new neurons in the ventral hippocampus, and **b** increased survival. *p < 0.05, ***p < 0.001. Error bars represent 1 SE

PPS resulted in a 46% decrease in cell proliferation in the suprapyramidal blade of the ventral dentate gyrus in males only (*F*_1,8.81_ = 4.9, *p* = 0.05, Fig. 2a), as measured by the number of co-labelled BrdU and DCX cells. There were no changes in the infrapyramidal blade or in females (*F*_1,7.541_ = 0.28, *p* = 0.62). Cell survival (as measured by DCX-labelled neurons) was increased by 65% in supra- and infrapyramidal blades of the ventral dentate gyrus after PPS in males (*F*_1,9.29_ = 14.21, *p* = 0.004, Fig. 2b) but not females (*F*_1,8.38_ = 1.25, *p* = 0.29). Volume of the dentate gyrus did not vary as a result of PPS (males: *F*_1,8.82_ = 0.01, *p* = 0.91; females: *F*_1,8.31_ = 0.09, *p* = 0.77).

### Behaviour

#### Pattern separation

**Fig. 3:**
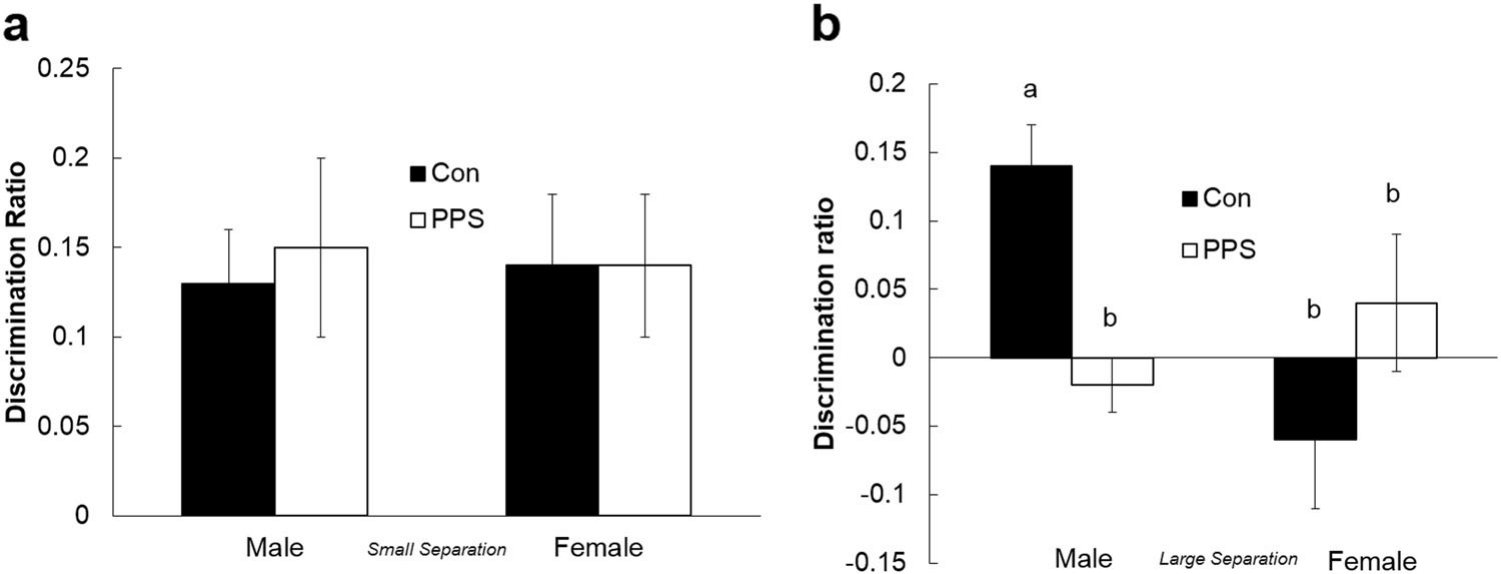
Pattern separation in control and PPS animals. **a** All animals successfully discriminated the object in the novel from the familiar spatial location at large separation distances, but **b** only control males were successful at small separation distances. Bars with different letters are significantly different to one another. Error bars represent 1SE

Animals from both groups spent an equal amount of time exploring all three objects in the sample phase (*F*_1,8.5_ = 0.01, *p* = 0.91). During the choice phase for LS, all animals spent more time exploring the novel object (A5) than the familiar one (A4) (one-sample *t* test: control males: *t* = 3.62, *p* = 0.005; PPS males: *t* = 3.05, *p* = 0.01; control females: *t* = 3.12, *p* = 0.01; PPS females: *t* = 3.18, *p* = 0.01), and both sexes were able to discriminate between objects equally well during LS (*F*_1,4.46_ = 0.07, *p* = 0.81, Fig. 3a), demonstrating that PPS did not affect pattern separation in the easy condition. During the choice phase for SS, only control males spent more time exploring the novel object than the familiar one (one-sample *t* test: control males: *t* = 4.38, = 0.001), and control males were the only group able to discriminate between novel and familiar objects in SS (*F*_1,37.55_ = 9.3, *p* = 0.006, Fig. 3b). Thus, control and stressed females were equally unable to distinguish between the novel and familiar objects in the SS condition, and stressed males showed an impairment in spatial pattern separation in the more difficult SS condition when compared to control males.

#### Fear conditioning: context

**Fig. 4:**
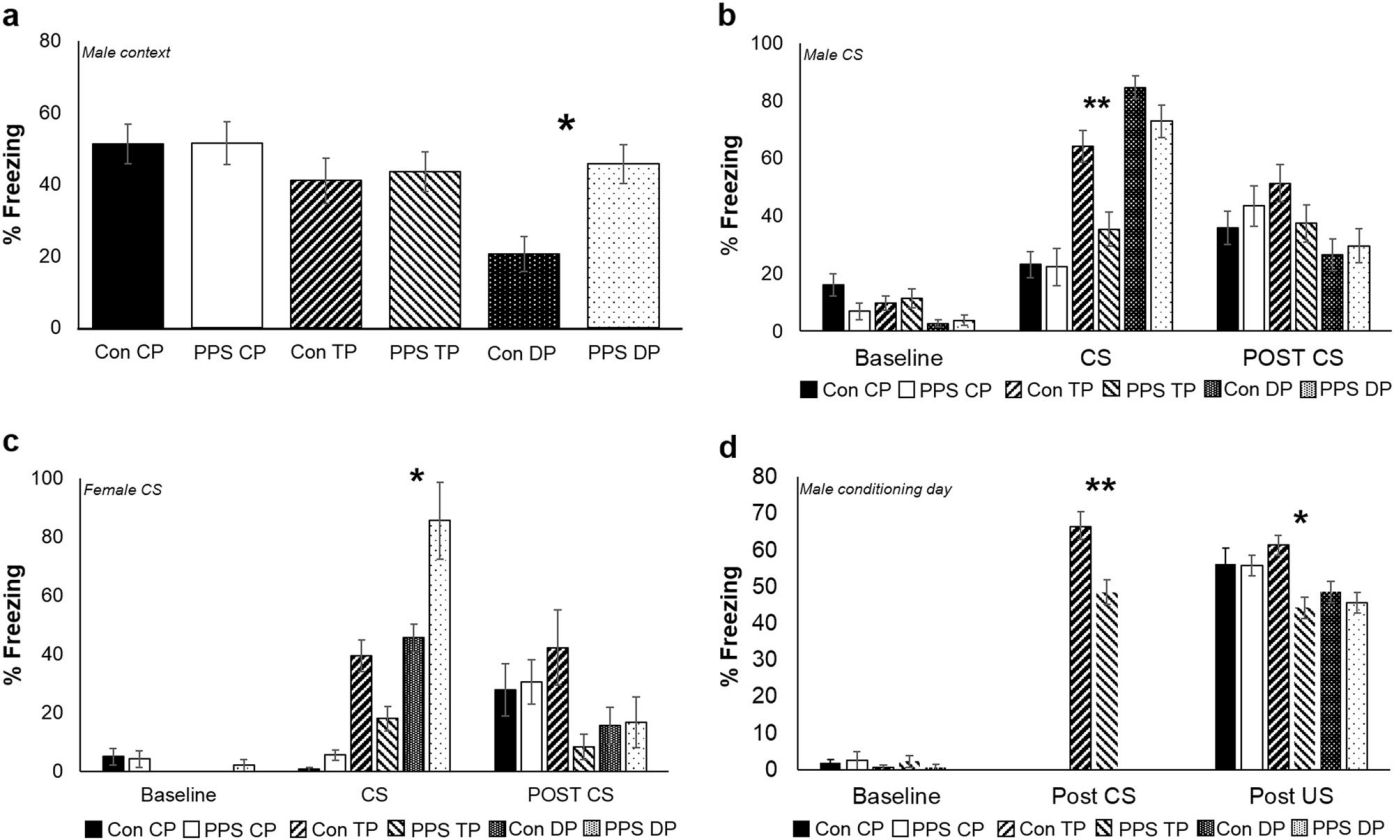
Fear responses in PPS and control animals exposed to three different conditioning protocols. **a** PPS males experiencing the delay protocol demonstrated enhanced levels of contextual freezing when compared to their control counterparts in a 24 h recall test. **b** PPS trace protocol males froze significantly less than controls to representation of the CS 48 h after conditioning. **c** PPS delay protocol females froze more than controls to representation of the CS 48 h after conditioning. **d** PPS males experiencing the trace protocol froze less post CS and post US on the conditioning day when compared to controls. Con control animals, PPS pre-pubertal stress animals, CP control protocol, TP trace protocol, DP delay protocol. *p < 0.05, **p < 0.01. Error bars represent 1SE

Results are shown from the first 90 s of each period only. Twenty-four hours after conditioning, male and female control animals experiencing CP and TP demonstrated robust levels of contextual freezing (Fig. 4a, data shown for males only). As expected, contextual freezing was markedly decreased in control DP animals where stronger associations to the CS are formed, resulting in decreased contextual freezing^39^. However, PPS resulted in increased contextual freezing in DP males (*F*_2,198.3_ = 3.24, *p* = 0.04, Fig. 4a) but not females (*F*_2,24.49_ = 0.67, *p* = 0.52). There were no differences between protocols or stressed and control animals in contextual freezing responses 72 h post conditioning (males: *F*_2,24.76_ = 0.75, *p* = 0.48; females: *F*_2,30.65_ = 2.12, *p* = 0.14).

#### Fear conditioning: cue recall

Forty-eight hours after conditioning cue recall was performed. In males, PPS reduced conditioned freezing during CS presentation in the TP (*F*_2,198.9_ = 3.6, *p* = 0.03, Fig. 4b). In females, PPS increased freezing during CS presentation in the DP (*F*_2,185_ = 3.37, *p* = 0.04, Fig. 4c). Overall, DP animals showed a greater freezing response to the CS than CP and TP animals, and TP froze more than CP animals (males: *F*_2,198.9=_ = 3.6, *p* < 0.0001; females: *F*_2,24.24_ = 25, *p* < 0.0001). This is expected as DP animals make stronger CS-US associations than TP animals, who make stronger associations than controls. There was no effect of PPS on post CS freezing, but overall, TP froze more than DP animals (males: *F*_2,197.1_ = 3.48, *p* = 0.03; females: *F*_2,27.08_ = 4.2, *p* = 0.03).

To investigate whether differences between the groups were present during encoding, we analysed freezing responses for 60 s after presentation of the 10 US (shocks) during conditioning, and during the 30 s ‘trace’ gap for TP animals (after presentation of CS, sound). All animals, males and females, froze more after US presentation than at baseline (*F*_10,631.1_ = 28.2, *p* < 0.0001), and TP animals froze more after CS presentation from CS 2 (*F*_10,222_ = 17.44, *p* < 0.0001). PPS resulted in lower levels of freezing in male TP animals both after US (*F*_1,11.14_ = 7.06, *p* = 0.02) and CS presentation (F_1,10.93_ = 10.74, *p* = 0.007, Fig. 4d).

### GABA and GABAergic interneurons

**Fig. 5:**
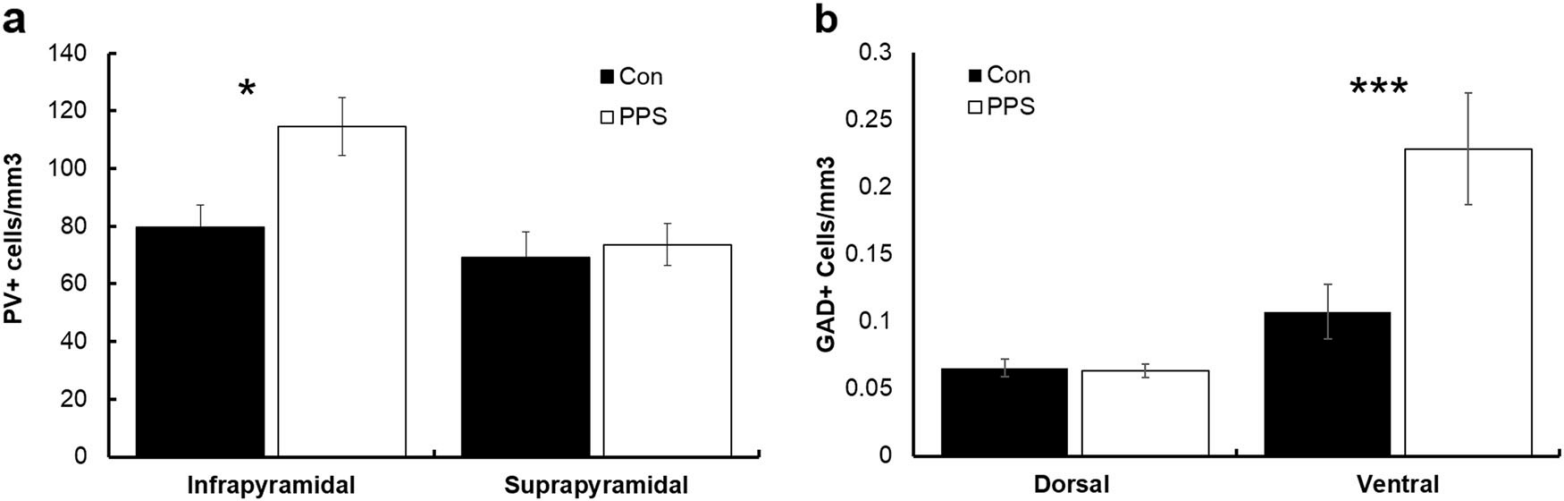
Effects of PPS on parvalbumin and GAD67 expression in the hippocampus. **a** Parvalbumin-positive cells (PV+) were increased in the ventral dentate gyrus after PPS in males, and **b** GAD67 (GAD+) was increased in the ventral hilus of PPS males. *p < 0.05, ***p < 0.001. Error bars represent 1 SE

PPS increased the expression of GABAergic (PV+) interneurons in the infrapyramidal but not suprapyramidal blade of the ventral hippocampus of males only (*F*_1,8.27_ = 6.83, *p* = 0.03, Fig. 5a). There were no differences in females (*F*_1,7.948_ = 3.2, *p* = 0.1). GAD67 was increased in the ventral but not dorsal hilus in stressed males (*F*_1,311_ = 18.3, *p* < 0.0001, Fig. 5b). Furthermore, following PPS, there was a significant positive correlation between the number of DCX+ and PV+ cells in the suprapyramidal blade of the dentate gyrus only (*r* = 0.64, *p* = 0.04).

## Discussion

### Summary

The present study demonstrates that PPS has a profound effect on the structure and function of the adult male hippocampus. Specifically, we found that PPS decreased production but enhanced survival of newborn neurons in the ventral dentate gyrus, and impaired pattern separation and trace fear conditioning—behaviours that depend on neurogenesis. We also found evidence of altered GABA signalling following PPS—expression of PV^+^ interneurons was increased in the ventral dentate gyrus and GAD67 increased in the ventral hilus. Due to GABA’s role in maintaining quiescence of neural progenitor cells and promoting survival of newborn neurons, this provides a potential molecular mechanism underlying alterations in AHN after PPS.

### Neurogenesis

We predicted that PPS would reduce proliferation (BrdU/DCX co-expressing cells) and survival (DCX only) of newborn neurons, as prenatal and early postnatal stressors (first 1–3 weeks of life) robustly decrease AHN in adult males^40,41^. In the present study, PPS decreased proliferation, but resulted in increased survival of newborn neurons, and these changes were specific to the ventral dentate gyrus in males. Thus, similar to prenatal and early postnatal stressors, PPS results in decreased cell proliferation in the male dentate gyrus^42,43^. However, the importance of timing is highlighted, as in contrast to other early-life stress paradigms, PPS increases cell survival. Delineating the contrasting mechanisms of dysfunction produced by stress at different early-life time points is crucial to producing effective interventions.

Altered neurogenesis is robustly associated with impairments in hippocampal-dependent behaviours, especially those with a temporal or spatial component^44^. Therefore, the second aim of the present study was to determine the consequences of PPS on two hippocampal-dependent behaviours, trace conditioning and pattern separation.

### Trace and delay fear conditioning

We previously demonstrated that PPS results in impaired hippocampal-dependent contextual fear conditioning responses in male animals^16^. Although an intact hippocampus is necessary for this task (at least in one trial studies)^45^, other brain regions, such as the amygdala, are also required^46,47^. Therefore, we used a trace/delay fear protocol to more precisely dissect the effects of PPS on the trace manipulation which is thought to rely on AHN.

Males subjected to PPS displayed lower conditioned fear responses to the CS but only after training with the trace protocol. This is likely due to deficits on encoding, as lower levels of freezing were observed in PPS trace protocol males after the second presentation of CS and US during training. Thus, PPS selectively impacts hippocampal function in males but not females. Hippocampal lesions impair responses to the CS in trace but not delay protocols, whereas other regions are equally important in both^47–49^. Furthermore, the acquisition of trace fear is impaired following ablation of AHN using opotogenetic or infusion methods, yet delay fear responses remain intact^28,45,50^. The relative contributions of dorsal and ventral hippocampus to trace conditioning are not entirely clear. One study found that bilateral infusions of the AMPA/kainate glutamate receptor antagonist CNQX into the dorsal dentate gyrus disrupts expression of trace fear conditioning (ventral inactivation was not assessed)^50^, whereas others reveal inactivation of the ventral, but not dorsal, hippocampus impairs acquisition and expression of trace fear^51^.

The effects of increased newborn neuronal survival on trace memories are unknown, but increased neurogenesis disrupts contextual fear memories^52,53^. Together, our data suggest that PPS-induced alterations in neurogenesis could underlie the impairments in trace fear conditioning observed in males in the present study.

Males who had experienced PPS froze more to contextual cues 24 h after training in the delay condition. The impact of PPS on contextual fear conditioning selectively in males is consistent with our previous findings, although here the direction of effect is different^16^. There are methodological differences between the studies which likely account for the disparity in results—a single presentation of the US in our previous work compared to the present study which used 10 CS-US pairings. Ablation of AHN results in impaired contextual fear responses in single but not multiple trial conditioning^54^. The enhanced contextual freezing in delay conditioning we measured may be modulated by other neural substrates including the amygdala, which is known to be overactive after multiple CS-US pairings in this model^34^. Females froze more to representation of the cue during the delay protocol only after PPS, another behaviour which relies more heavily on the amygdala^55^.

### Pattern separation

AHN is necessary for successful spatial pattern separation, particularly when stimuli are of increasing similarity^56^. We showed that animals were able to discriminate between stimuli when they were presented at large separation distances, but only control males successfully completed the task at the smaller, more difficult, separation distances. Deficits in pattern separation are particularly evident in psychiatric disorders with a strong hippocampal phenotype, such as post-traumatic stress disorder, schizophrenia and major depressive disorder^57^. To date, no studies have considered the impact of early-life stress on pattern separation. This study provides novel evidence for the role of PPS in disrupted pattern separation ability in adult life, and again points to a role between disrupted AHN and impaired hippocampal-dependent behaviour.

Typically, the dorsal hippocampus has been associated with impairments in cognitive and spatial behaviour (such as pattern separation) and the ventral hippocampus with emotional responses (such as those associated with fear conditioning)^44^. However, the situation is likely more complex, and the dorsal hippocampus has been shown to play a role in trace fear responses, especially with long trace intervals^58^. Presently, the relative contributions of dorsal and ventral hippocampus to the pattern separation task used in the present study are unknown. However, studies using pattern separation for reward value suggest a crucial role for the ventral dentate gyrus^59^, and one study found expression of the immediate-early gene *zif*268 in both ventral and dorsal dentate gyrus when rats were required to change between different navigational strategies in a plus maze^60^. Further work is required to delineate the relative contributions of dorsal and ventral hippocampus to the pattern separation task used in the current study. Here, we see impairments in both emotional (trace fear) and spatial (pattern separation) tasks correlated with altered neurogenesis in the ventral hippocampus.

### GABAergic interneurons

Neural stem cells, the substate for neurogenesis, are largely quiescent, a state maintained through tonic GABA current from PV (PV+) interneurons^61^. We found that PPS increased PV+ interneurons in the ventral dentate gyrus, and increased GAD67, the rate-limiting enzyme in GABA production, in the ventral hilus in males specifically. This suggests a mechanism whereby GABA could be responsible for supressing generation of adult-born neurons in the ventral hippocampus in males following PPS.

GABA promotes survival of newborn neurons^61,62^. This may explain why although generation of new neurons was decreased, survival of DCX-expressing neurons, a population aged 24 h–2 weeks, was markedly increased after PPS in the male ventral dentate gyrus, especially as we find a positive correlation between the number of PV+ and DCX+ cells in the ventral dentate gyrus. Taken together, altered GABA signalling through increased numbers of PV+ interneurons may underlie aberrant neurogenesis and behaviour observed after PPS. Altered GABA signalling has been previously found in models of PPS, with levels of α1–3 subunits of the ionotropic GABA A receptor altered in a region-specific manner^63^.

### Sex differences

Sex-based differences in the development of neuropsychiatric disorders abound, and have been found previously in our PPS model^16,64,65^. In the present study, PPS produces fewer changes in females than males. Specifically, PPS enhanced freezing to representation of the CS after a delay protocol only in females, with no other changes evident. This complements research by Toledo-Rodriguez and Sandi^66^, who found that PPS on PND 28–30 impaired extinction to a tone CS in adult males but not females. Interestingly, this study found increased fear responses to a tone in adolescent males, and reduced contextual freezing in adolescent females after PPS, highlighting the importance of timing when assessing the long-term outcomes of early-life stress.

PPS decreased the production yet increased the survival of newborn neurons in the infrapyramidal blade of the ventral dentate gyrus of males, with no effect in females. This mirrors the adult situation, where males demonstrate greater sensitivity to the effects of stress hormones, showing more extensive suppression of neural stem cell proliferation in the dentate gyrus^67^. However, adults also show decreased survival of newborn neurons in response to stress, which is in contrast to the increased survival observed in the present study. Prenatal stressors almost universally result in decreased generation and survival of newborn neurons in both males and females, whereas postnatal stress in the first 1–2 weeks of life produces more variable results^40^. Age of assessment is also vital: males experiencing early postnatal stress tend to have enhanced neurogenesis at PND 21, which switches to suppression as the animals age (as measured by DCX-positive cells). In contrast, females display decreased neurogenesis at PND 21, and this effect disappears in adulthood (DCX-positive cells). Less is known about the age- and sex-specific effects of this early prenatal stress on cell proliferation. Our study demonstrates that PPS produces a decrease in proliferation (measured by BrdU) with an increase in survival (DCX-positive cells) in adult males, with no effects apparent in females, marking the pre-pubertal phase as a time-point with unique consequences for AHN.

Sex differences were also seen in the pattern separation task, male and female rats performed equally well when the task was easy, but only control males were able to complete the difficult phase. We previously found male animals were superior in a spatial navigation task (water maze)^16^, and a number of other studies reveal a male advantage in spatial reference; however, others find no difference^68^. The fact that control females could not complete the more difficult pattern separation means assessing the effects of PPS on females in this task was not possible, and alternative tasks are needed to probe further female responses to PPS.

Expression of PV+, GABAergic interneurons and GAD67 were altered in males experiencing PPS, but remained unchanged in females. Prenatal stress decreases PV+ interneurons and GAD67 expression in the frontal cortex and hippocampus in early and adult life in males^69,70^, and alters the expression of GABA receptor subunits in a region- and sex-dependent manner^71^. PPS has previously been shown to decrease the expression of GABA_A_ receptor α1 subunit in the hippocampus of adult males, and elevation is associated with stress resilience^72,73^, but little is known of the effects in females. Exposure to a more extended PPS paradigm (spanning PND 28–42) resulted in reduced messenger RNA levels of GAD67 in the central nucleus of the amygdala in male rats^74^, whereas a shorter protocol (PND 28–30) was insufficient to elicit such changes. This suggests that the exact timing and duration of early-life stress are likely crucial in determining later outcomes. Our results add to the literature demonstrating sex differences in response to PPS. In the present study, males display greater responses to PPS, but previous work demonstrates that females are sensitive in different ways. For example, females display learning impairments, exhibit greater compulsivity in a delay discounting task and have reduced preference for sweet solutions (anhedonia)^75,76^. Furthermore, males and females react similarly regarding certain behaviours: both sexes display heightened anxiety and altered cognitive bias after PPS^17,33,77^. In humans sex differences are also observed: associations between childhood maltreatment and incidents of anxiety and depression are higher in women than men, whereas risk for substance use disorder show the opposite pattern^78,79^. This suggests that males and females are distinct in their vulnerabilities to the experience of stress during development. Understanding these differences is crucial in the advancement of appropriate interventions and treatments for psychiatric illness associated with experience of childhood maltreatment.
